# SET-NUP214融合基因阳性儿童急性髓系白血病二例

**DOI:** 10.3760/cma.j.issn.0253-2727.2021.09.011

**Published:** 2021-09

**Authors:** 湧智 郑, 晶晶 温, 凌燕 王, 浩 郑, 雪玲 华, 健 李, 建达 胡

**Affiliations:** 福建医科大学附属协和医院，福建省血液病研究所，福建省血液病学重点实验室，福州 350001 Department of Hematology, Fujian Medical University Union Hospital, Fujian Institute of Hematology, Fujian Provincial Key Laboratory on Hematology, Fuzhou 350001, China

例1，男，12岁，于2018年10月以“发热10 d”为主诉入院。血常规：WBC 231.8×10^9^/L，幼稚细胞96％，HGB 92 g/L，PLT 39×10^9^/L；骨髓象：急性粒细胞白血病部分分化型；免疫分型：异常髓系原始细胞占有核细胞的97.73％，表达CD7、CD34、CD9、CD123、CD33，部分表达CD38、MPO、CD56，弱表达CD11b、CD117、CD13，符合急性髓系白血病（AML）表型，伴CD56、CD7表达；FISH（PDGFRB、CRLF2、ABL1、ABL2、CSF1R）提示：ABL1分离探针存在分离信号（即存在9q34断裂缺失），阳性率95％（图1A）；白血病融合基因筛查：SET-NUP214融合基因阳性；一代测序证实SET基因7号外显子与NUP214基因18外显子发生融合；染色体核型为46,XY[9]。诊断：SET-NUP214融合基因阳性AML。予小剂量阿糖胞苷（100 mg·m^−2^·d^−1^）治疗3 d，WBC降至30×10^9^/L以下，予FLAG-IDA方案（氟达拉滨30 mg/m^2^，第2～6天；阿糖胞苷2 g/m^2^，第2～6天；G-CSF 5 µg/m^2^，第1～7天；去甲氧柔红霉素8 mg/m^2^，第4～6天）诱导治疗。1个月后复查骨髓：有核细胞减低，未见原始幼稚细胞；流式细胞术检测微小残留病（MRD）0.36％ ；SET-NUP214融合基因定量0.113％，FISH（ABL1重排）阴性。再予FLAG-IDA化疗1个疗程，复查骨髓：完全缓解（CR）；MRD、SET-NUP214融合基因定量、FISH（ABL1重排）均阴性。2019年3月行异基因造血干细胞移植（allo-HSCT）（单倍型，姐供弟，A供B；预处理方案为FA5BuCy+ATG）。移植后第22天中性粒细胞植入，移植后3、6、9个月复查骨髓均处于CR状态，但血小板始终不能恢复正常，多次STR检测均提示混合嵌合状态，考虑植入不良，予减停抗GVHD药物、输注间充质干细胞等处理，仍不能达到完全植入，在移植后11个月（距诱导化疗15.5个月）骨髓复发，1个月后死亡。

例2，男，10岁，于2018年11月以“发热、腹痛6 d，面色苍白4 d”为主诉入院。血常规：WBC 38.75×10^9^/L，幼稚细胞占86％，HGB 47 g/L，PLT 34×10^9^/L；骨髓象：急性粒细胞白血病微分化型。流式细胞术检测：异常髓系原始细胞占有核细胞94.16％，表达CD34、CD117、CD7、CD38、CD123、CD33，部分表达CD11b、CD13、TdT，符合AML表型伴CD7表达；FISH：ABL1分离探针存在分离信号（存在9q34断裂缺失），阳性率96％（图1B）；白血病43中融合基因筛查：SET-NUP214融合基因阳性；一代测序证实SET基因7号外显子与NUP214基因18外显子发生融合；染色体核型为46,XY,del（5）（?q31）,add（6）（p21）,del（6）（p23）,inc[10]。故诊断为SET-NUP214融合基因阳性AML。予FLAG-IDA方案诱导化疗1个疗程，复查骨髓CR，MRD 0.09％，SET-CAN融合基因定量0.11％，FISH（ABL1重排）阴性；再予FLAG-IDA方案化疗1个疗程，复查骨髓CR；MRD、SET-NUP214融合基因定量、FISH（ABL1重排）均阴性。2019年2月行allo-HSCT（全相合，姐供弟，A供A；预处理方案同例1），移植后第24天中性粒细胞植入，第35天血小板植入，STR 100％。随访至移植后24个月，患儿已停抗GVHD药物，仍处于CR状态。

**图1:**
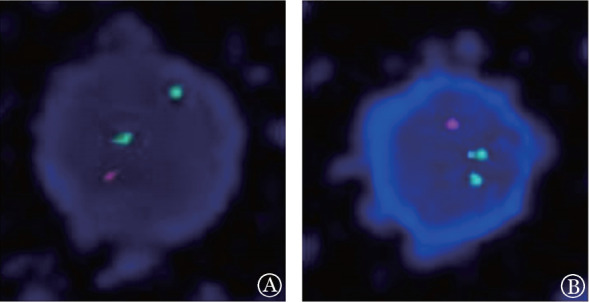
例1（A）和例2（B）的FISH结果

